# Adaptive mechanisms in no flow vs. low flow ischemia in equine jejunum epithelium: Different paths to the same destination

**DOI:** 10.3389/fvets.2022.947482

**Published:** 2022-09-08

**Authors:** Franziska Dengler, Felix Sternberg, Marei Grages, Sabine BR Kästner, Nicole Verhaar

**Affiliations:** ^1^Department of Biochemical Sciences, Institute of Physiology, Pathophysiology and Biophysics, University of Veterinary Medicine, Vienna, Austria; ^2^Clinic for Horses, University of Veterinary Medicine Hannover, Hannover, Germany; ^3^Small Animal Clinic, University of Veterinary Medicine Hannover, Hannover, Germany

**Keywords:** AMPK, autophagy, HIF1α, ischemia-reperfusion injury, LC3, mitophagy, NRF2, ROS

## Abstract

Intestinal ischemia reperfusion injury (IRI) is a frequent complication of equine colic. Several mechanisms may be involved in adaptation of the intestinal epithelium to IRI and might infer therapeutic potential, including hypoxia-inducible factor (HIF) 1α, AMP-activated protein kinase (AMPK), nuclear factor-erythroid 2-related factor 2 (NRF2), and induction of autophagy. However, the mechanisms supporting adaptation and thus cellular survival are not completely understood yet. We investigated the activation of specific adaptation mechanisms in both no and low flow ischemia and reperfusion simulated in equine jejunum epithelium *in vivo*. We found an activation of HIF1α in no and low flow ischemia as indicated by increased levels of HIF1α target genes and phosphorylation of AMPKα tended to increase during ischemia. Furthermore, the protein expression of the autophagy marker LC3B in combination with decreased expression of nuclear-encoded mitochondrial genes indicates an increased rate of mitophagy in equine intestinal IRI, possibly preventing damage by mitochondria-derived reactive oxygen species (ROS). Interestingly, ROS levels were increased only shortly after the onset of low flow ischemia, which may be explained by an increased antioxidative defense, although NFR2 was not activated in this setup. In conclusion, we could demonstrate that a variety of adaptation mechanisms manipulating different aspects of cellular homeostasis are activated in IRI irrespective of the ischemia model, and that mitophagy might be an important factor for epithelial survival following small intestinal ischemia in horses that should be investigated further.

## Introduction

Intestinal ischemia reperfusion injury (IRI) plays a central role in the pathogenesis of equine colic (1). Strangulating hernias, intestinal volvulus, pedunculated lipoma, or arterial embolism as a result of parasites can lead to the occlusion of the mesenterial vessels (2, 3). This results in the discontinuation of oxygen and nutrient supply to the intestinal wall. While the initial ischemic insult already puts the tissue at risk, resolving the ischemia, i.e., reperfusion, paradoxically even adds to the critical situation by challenging cellular redox homeostasis (4), and may result in irreversible epithelial damage. Clinically, the patients show signs of severe pain, and the prognosis may be guarded: up to 50% of horses referred for signs of colic to a tertiary hospital do not survive to discharge (5, 6). Even though severely damaged tissues are usually resected, sections of bowel with mild ischemic injury that appear to be viable after repositioning are often preserved. Furthermore, we know that the intestine peripheral to the strangulating lesion undergoes changes as well (7, 8). It is unclear if this is the result of the ischemia itself, reperfusion injury, or a combination of both. Nevertheless, it emphasizes the importance of investigating the cellular mechanisms of intestinal IRI over time.

The intestinal epithelium is most sensitive to IRI (9, 10) and thus its integrity is decisive for the fate of the patient. As the barrier between the gut lumen and the rest of the organism, it is a critical factor for the systemic health of the whole organism and thus the outcome of intestinal IRI. Damage to this barrier can result in endotoxemia and sepsis (11). Cells have elaborate mechanisms to prevent an uncontrolled disruption of the epithelial layer and to adapt to challenges like IRI (12), as demonstrated by the protective effect of ischemic pre- and even postconditioning (10, 13–16). Directly targeted, these mechanisms could offer new therapeutic approaches for strangulating intestinal diseases in horses. So far, the mechanisms mediating the adaptation have hardly been investigated in equine intestinal epithelium. Hence, we aimed to examine the involvement of the major pathways suspected to mediate an adaptation to ischemia based on findings in other species and tissues in equine intestinal epithelium and to identify promising therapeutic targets in this study.

Although clinical data on blood flow during intestinal strangulation are lacking, it is assumed that the occlusion of the mesenterial vessels is not always complete. Likewise, in experimental ischemia/reperfusion studies either a no or a low flow model is applied, i.e., a complete or partial occlusion of arterial blood flow. The resulting differences in oxygenation, efflux of metabolites and impact of reperfusion could also affect the adaptation mechanisms that are activated. Therefore, we compared both no and low flow ischemia for the activation of the pathways described hereinafter.

Hypoxia inducible factor (HIF) 1α is well-known as the major regulator of hypoxic adaptation (17, 18) and may also be crucial for adaptation of the equine intestinal epithelium, but its activation in equine intestinal ischemia is uncertain (19, 20). Additional pathways may mediate an even faster adaptation to ensure epithelial survival. AMP-activated protein kinase (AMPK) is a major candidate for this quick adaptation. Originally characterized as a cellular energy sensor (21, 22), AMPK has also been shown to be activated in hypoxia (23, 24), in the ischemic heart (25), and intestine (26–28) in rodents and human cell lines. An activation of AMPK would subsequently stabilize intracellular energy levels and thereby support epithelial survival (21). To ensure cellular energy supply, AMPK also activates macroautophagy, i.e., a “self-digestion” of the cells, including the specific degradation of damaged cell organelles such as mitochondria, i.e., mitophagy (22, 29, 30). Autophagy is a constantly occurring process to recycle misfolded or aged proteins or xenogens, but also to mobilize metabolic substrates during starvation (31). Recent studies proposed that the grade of autophagy might be crucial for the cellular survival in the course of IRI (31–35). However, there are controversial results whether autophagy is beneficial or detrimental in IRI (9, 36–40). Due to the complete lack of nutrients, an activation of AMPK and thus autophagy is more likely to happen (faster) in no flow ischemia, whereas in low flow conditions the activation of AMPK may be postponed or completely abolished, possibly causing differing rates of autophagy under the different conditions.

An important difference between no and low flow ischemia is the potential formation of reactive oxygen species (ROS). No flow conditions accompanied by a complete lack of oxygen may lead to exacerbated ROS formation in the reperfusion phase, while the decreased oxygen levels still reaching the tissue during low flow ischemia may immediately lead to ROS formation by damaged mitochondria (41). Thus, besides ensuring cellular energy supply, antioxidative mechanisms are also important for cellular survival. Nuclear factor-erythroid 2-related factor 2 (NRF2) has been described as a transcription factor mediating the upregulation of mechanisms such as superoxide-dismutase (SOD) to scavenge ROS and thereby protect the cells (41). Given the higher potential of ROS production in low flow ischemia, an activation of NRF2 is to be expected, yet this has never been investigated comparatively before.

To identify the therapeutic potential of these pathways, we investigated the activation of autophagy and of the central adaptation mechanisms (HIF1α, AMPK, NRF2) during the course of ischemia and reperfusion to obtain a timeline of their activation. Additionally, we simulated both no and low flow ischemia *in vivo* in equine jejunum epithelium to compare both ischemia models for differential regulation of these pathways. We hypothesized that no flow ischemia induces a quicker and more pronounced activation of the HIF1α and AMPK pathway due to the complete lack of oxygen and nutrients, whereas the possibly higher amounts of ROS formed in low flow ischemia rather activate antioxidative pathways such as NRF2.

## Materials and methods

### Animals and ischemia reperfusion injury

The study was reviewed by the Ethics Committee for Animal Experiments of Lower Saxony, Germany, and approved according to Section 8 of the German Animal Welfare Act (LAVES 33.8-42502-04-19/3240). Thirteen horses, owned by the University of Veterinary Medicine Hannover, were divided into two groups using simple randomization with an equal allocation ratio. One group (*N* = 7) underwent no flow ischemia (i.e., 100% occlusion of the mesenteric arteries) while in another group (*N* = 6) low flow ischemia was applied [i.e., occlusion of 80% of the mesenteric blood flow (42, 43)].

In two horses of the low flow group the blood flow in the mesenteric vessels was not consistently lowered to the desired 80% as the analysis of blood flow measurements revealed after the procedure. Consequently, it was decided to preclude these animals from this analysis. The remaining 11 warmblood horses had a mean age of 16 ± 8 years and weighed 538 ± 67 kg. The animals consisted of 6 females and 5 males (3 geldings, 2 stallions), and there were no differences in demographics between the groups. All horses were systemically healthy as determined by physical examination, complete blood count, and fecal examination. They had been elected for euthanasia due to chronic musculoskeletal problems. The horses were stabled at the facilities of the equine clinic of the University of Veterinary Medicine Hannover at least 2 weeks prior to surgery. No medication was administered during this time. The horses had free access to hay and water and were hand walked daily. Prior to anesthesia, feed but not water was withheld for 6 h.

After premedication with 5 μg/kg dexmedetomidine (Dexdomitor, Orion Corporation, Espoo, Finland), general anesthesia was induced with 0.05 mg/kg diazepam (Diazedor, WDT eG, Garbsen, Germany) and 2.2 mg/kg ketamine (Narketan, Vétoquinol GmbH, Ismaning, Germany). Anesthesia was maintained with isoflurane (Isofluran CP, CP-Pharma GmbH, Burgdorf, Germany) in 100% oxygen and a continuous rate infusion with dexmedetomidine (5 μg/kg/h). Lactated Ringer's solution (Ringer-Laktat EcobagClick, B. Braun Melsungen AG, Melsungen, Germany) and dobutamine (Dobutamin-ratiopharm 250 mg, Ratiopharm GmbH, Ulm, Germany) were administered to effect, to maintain the mean arterial blood pressure between 60 and 80 mmHg.

A routine pre-umbilical median laparotomy was performed in dorsal recumbency. Segmental small intestinal ischemia was induced in 2 m jejunum by occlusion of the mesenteric arteries and veins with umbilical tape. In the horses subjected to no flow ischemia, this was complemented by the additional occlusion of each jejunal vessel with a hemostatic clamp to ensure 100% blood flow reduction. The low flow ischemia was induced by tightening the umbilical tape under monitoring of the intestinal microperfusion with microlightguide spectrophotometry and laser Doppler fluxmetry (O2C, LEA Medizintechnik GmbH, Giessen, Germany), until a reduction of 80% of the blood flow could be confirmed (44). The ischemia was maintained for 120 min, followed by 120 min of reperfusion. Subsequently, the horses were euthanized by intravenous administration of 90 mg/kg pentobarbital (Release 50 mg/ml, WDT eG, Garbsen, Germany) without regaining consciousness.

Full thickness intestinal segments of 15 cm jejunum were taken just before induction of ischemia (control 1, C1), after 1 h and after 2 h of ischemia (ischemia samples, I1 and I2), after 1 h and 2 h of reperfusion (reperfusion samples, R1 and R2). Parallel to sample R2, an additional intestinal sample was taken approximately 5 m orally of the segment undergoing IRI (C2). After sampling, the lumen of the remaining intestinal segments was occluded with umbilical tape and lavaged prior to replacement in the abdominal cavity. The samples were rinsed in 4°C PBS immediately and the intestinal mucosa was separated mechanically from the underlying muscle and snap frozen in liquid nitrogen. Then it was stored at −80°C until further processing.

### Two-step RT-qPCR

Total RNA was isolated from 20 mg of the tissue homogenized in lysis buffer from the ReliaPrep™ RNA Miniprep System (Promega GmbH, Mannheim, Germany) using a FastPrep 24-5G (MP Biomedicals, Eschwege, Germany). Then, the samples were further processed according to the manufacturer's protocol including treatment with DNase. The RNA concentration and quality were determined using a spectrophotometer (DeNovix DS-11, Wilmington, USA). One microgram of high-quality RNA was used for cDNA synthesis using the GoScript^TM^ Reverse Transcriptase Kit (Promega, Mannheim, Germany) according to the manufacturer's instructions using a GeneAmp 9700 PCR System (Applied Biosystems, Thermo Fisher Scientific, Vienna, Austria).

For qPCR, the resulting cDNA was diluted 1:20 and 1 μl was used in a 10 μl reaction volume containing a ready-to-use premix of SYBR Green I dye, dNTPs, stabilizers, and enhancers (GoTaq^®^, Promega GmbH, Mannheim, Germany), 112 nM primer mix and DNase-free water. These mixtures were pipetted in 384-well plates (# 04-083-0384, nerbe plus, Winsen, Germany) and processed in a qTOWER3 84 (Analytik Jena GmbH, Jena, Germany). A no template control (NTC) with DNase-free water instead of cDNA was applied for each run. qPCR reactions for each sample and gene were run in duplicate to minimize dispensation artifacts. The PCR cycles were run using automatic fluorescence emission following each PCR cycle, and the amplification specificity was checked after each run by melting curve analysis. The primer sequences for qPCR are shown in Table 1; the denaturation temperature was always 95°C and extension and annealing were performed at 60°C. The primers were designed with the Primer BLAST tool from the National Center for Biotechnology Information (NCBI, Bethesda, MD, USA) according to known sequences from the Basic Local Alignment Search Tool (BLAST) in the gene bank database of the NCBI and synthesized by Eurofins MWG (Ebersberg, Germany). The quantification cycle was determined using the qPCRsoft384 1.2 software (Analytik Jena GmbH, Jena, Germany). The C_t_ values set by the software were applied after checking them optically. For analysis of the data, the ΔΔC_t_ method was used to compare the mRNA expression.

**Table 1:** Primers used for RT-qPCR.

**Gene name**	**Gene bank accession no**.	**Primer sequence** **(5^′^-3^′^)**	**Amplicon length** **(bp)**
*ALDOA*	XM_023616037.1	F: CTCTGGCCTATTCCTTTACCCC	101
		R: TGGTGGCAGTGAGTTCCTTTCC	
*BNIP3*	XM_023636878.1	F: GCCATCGGATTGGGGATCTA	113
		R: TGCGAGCTCCGATACACATC	
*G6PD*	XM_023634095.1	F: CGAGCCCTTCTTCAAAGCCA	98
		R: GAGGCTGAGTCGTCATACTGG	
*GLUT1*	NM_001163971.2	F: TACGTGGAGCAACTCTGTGG	112
		R: AATCTCATCGAAGGTCCGGC	
*GPX1*	NM_001166479.1	F: AGTTCGGGCATCAGGAGAAC	93
		R: AGTGTGAAGTTGGGCTCGAA	
*HK2*	XM_023618374.1	F: CAGACGGGACAGAACATGG	93
		R: TGAAGCCCGTTGTCCGTTAC	
*HPRT1*	XM_023634464.1	F: GGGATTTGAATCACGTTTGTGTC	94
		R: CTCCAGATGTTTCTCCAACTCAACC	
*NQO1*	XM_005608375.3	F: CTGGCCAATTCAGAGTGGCA	96
		R: TCGGGTATCCTTGGGAAGGT	
*NRF2*	XM_023622057.1	F: CCAACTTCTCCCAGGTTTCCC	89
		R: CAAACGGGAATGTCTCTGCCA	
*PRDX3*	XM_001493616.5	F: AACACCCCACGGAAGAATGG	94
		R: AACAGCACACCGTAGTCTCG	
*PRDX5*	XM_023654308.1	F: ACACGAAAGGCAAGGTTCGG	104
		R: GTCGGTTCCCAAAGAGGGAC	
*PPIA*	XM_001496943.5	F: GCCAAGACTGAGTGGTTGGAT	112
		R: TTGCTGGTCTTGCCATTCCT	
*SOD1*	NM_001081826.3	F: CCCGTCGTTCTGAAGGGATT	98
		R: AGCAGTGGTACAGCCTTGTG	
*SOD2*	NM_001082517.2	F: GTTGGGGTTGGCTTGGTTTC	103
		R: CAGCAGGGGAATAAGACCTGT	
*TXN2*	XM_023631871.1	F: CCATAGAGTATGAGGTGTCCGC	92
		R: GGTCTTCATCCTTGATGCCCA	

Two horses from the no flow group were excluded from this analysis as qPCR data indicated a low efficiency of replication, indicating the presence of PCR inhibitors in these samples. To ensure correct statistical comparison, we excluded these horses from the gene expression analysis completely as single time points would be missing.

Normalization of the samples was achieved using the same amounts of RNA for processing and by normalizing the data for the target genes with the aid of the reference genes hypoxanthine guanine phosphoribosyltransferase 1 (HPRT1) and peptidylprolyl-isomerase A (PPIA). Therefore, the geometric mean of both reference genes' C_t_ values was calculated for each sample and used for normalization. The reference genes have been proven to be stable under the experimental conditions applied in our study. Their stability was tested using the program RefFinder (45).

In order to assess an activation of HIF1α, we measured the mRNA expression levels of known target genes: glucose transporter 1 (*GLUT1*), aldolase A (*ALDOA*), and hexokinase (*HK*) 2. Besides HIF1α, another transcription factor that might play an important role in IRI is the master regulator of antioxidative defense NRF2. Therefore, we measured the mRNA expression of *NRF2* and its target genes NAD(P)H quinone dehydrogenase 1 (*NQO1)*, glucose-6-phosphate dehydrogenase *(G6PD)*, and *SOD1*. To assess mitochondrial homeostasis, we measured the mRNA expression of mitochondrial markers, i.e., BCL2 interacting protein 3 (*BNIP3)*, glutathione peroxidase 1 *(GPX1)*, peroxiredoxin *(PRDX) 3* and *5*, thioredoxin 2 *(TXN2)*, and *SOD2*.

### Western blot

For total protein extraction, 100 mg of the tissue were homogenized in RIPA buffer using a FastPrep 24-5G (MP Biomedicals, Eschwege, Germany) with a protease and phosphatase inhibitor (cOmpleteTM Ultra Tablets, Roche Austria, Vienna, Austria). The protein concentration was measured with the EnSpire Multimode Plate Reader (PerkinElmer LAS -GmbH, Rodgau, Germany) using the bicinchoninic acid method and bovine serum albumin (BSA) as standard. As two samples from one horse of the no flow group did not contain sufficient amounts of tissue for extraction of total protein, this horse was omitted from the protein analyses.

For western blot analysis, the protein samples were separated by sodium dodecyl sulfate-polyacrylamide gel electrophoresis (SDS-PAGE) using 20 μg protein/well. Subsequently, the samples were transferred onto a nitrocellulose membrane (Nitropure 0.45 μm, Osmonics, Westborough, USA) using the EasyPhor Semi Dry Blotter (Biozym, Vienna, Austria). The membrane was preincubated in 2% BSA in TRIS-buffered saline containing 0.2% Tween-20 (TBST) for 1 h. Then it was incubated with the primary antibodies (see Table 2) at 4°C over night with gentle agitation. After washing with TBST, the membranes were incubated with an HRP-coupled secondary antibody (see Table 2) at room temperature with gentle agitation for 1 h. Subsequently, the membranes were rinsed again with TBST, then the signal was detected by enhanced chemiluminescence using a ChemiDoc IT 600 Imaging System (UVP/Analytik Jena GmbH, Jena, Germany) and analyzed with the VisionWorks^TM^ software (Analytik Jena GmbH, Jena, Germany). β-ACTIN was used as loading control for normalization of each quantitative blot. For the detection of total and phospho-AMPKα, the membranes were first incubated with anti-pAMPKα-antibody; after detection of the signal, they were stripped with a Na-citrate buffer (pH 2.2) and subsequently the protocol was repeated for total AMPKα beginning with the preincubation in 2% BSA. An increased ratio of pAMPKα to AMPKα indicates an activation of AMPK. Furthermore, we evaluated the protein levels of NRF2 and to assess the magnitude of autophagy we measured the amount of lipidated LC3, i.e., LC3II.

**Table 2:** Antibodies used for western blot.

**Target**	**Primary** **antibody**	**Manufacturer/** **catalog** **number**	**Dilution**	**Secondary** **antibody**	**Manufacturer/** **catalog number**	**Dilution**
β-ACTIN	Mouse-anti-β-Actin	sigma aldrich/A5441	1:5000	Goat-anti-mouse HRP	Merck Millipore (Darmstadt, Germany)/AP181P	1:5000
AMPKα	Rabbit-anti-AMPKα	Cellsignalling (Frankfurt, Germany)/#5831	1:1000	Donkey-anti-rabbit HRP	Cellsignalling/#7074	1:10000
pAMPKα	Rabbit-anti-pAMPKα (Thr172)	Cellsignalling/#2535	1:1000	Donkey-anti-rabbit HRP	Cellsignalling/#7074	1:10000
LC3B	Rabbit-anti-LC3B	Novus Biologicals (Abingdon, UK)/NB100-2220	1:1000	Donkey-anti-rabbit HRP	Cellsignalling/#7074	1:10000
NRF2	Rabbit-anti-NRF2	Proteintech (Rosemont, USA)/16396-1-AP	1:500	Donkey-anti-rabbit HRP	Cellsignalling/#7074	1:10000

### Measurement of reactive oxygen species

ROS were measured using 2,7-dichlorofluorescein diacetate (DCFDA, Sigma Aldrich, Darmstadt, Germany) as described previously (46, 47). 100 mg of the tissue were homogenized in 1 ml Tris-HCl buffer (40 mM, pH 7.4) using a FastPrep 24-5G (MP Biomedicals, Eschwege, Germany). After a short centrifugation, 100 μl of the homogenate were incubated with 1 ml of a 50 nM DCFDA solution in TrisHCl at 37°C for 40 min. To check for tissue autofluorescence, 100 μl of the homogenate were incubated with 1 ml TrisHCl under the same conditions. Then, triplicates of 150 μl each were measured in black 96-well plates in an EnSpire Multimode Plate Reader (PerkinElmer LAS GmbH, Rodgau, Germany) at an excitation at λ = 485 nm and an emission at λ = 525 nm. The results were normalized to the protein content of each sample. The protein concentration was measured as described above.

### Statistics

The results are depicted as boxplots showing the median + 10th, 25th, 75th, and 90th percentiles. The significance is expressed as the probability of error (p). The data of the technical replicates were pooled for each time point and animal (N) for statistical analysis and were expressed relative to C1 of the same animal. The data were checked for normality and equal variance using a Shapiro-Wilk and Brown-Forsythe test, respectively. Statistical analysis was performed with SigmaPlot 14.5 (Systat Software, Erkrath, Germany) using a two-way repeated measurements (RM) ANOVA for one independent effect (ischemia model) and the time points as repeated effect for each animal. A *post hoc* Holm-Sidak test was performed for the multiple comparisons between the time points (I1, I2, R1, R2, C2), as well as between the different ischemia models (no/low flow). The differences were assumed to be statistically significant if p < 0.05.

## Results

### RT-qPCR

**Figure 1:**
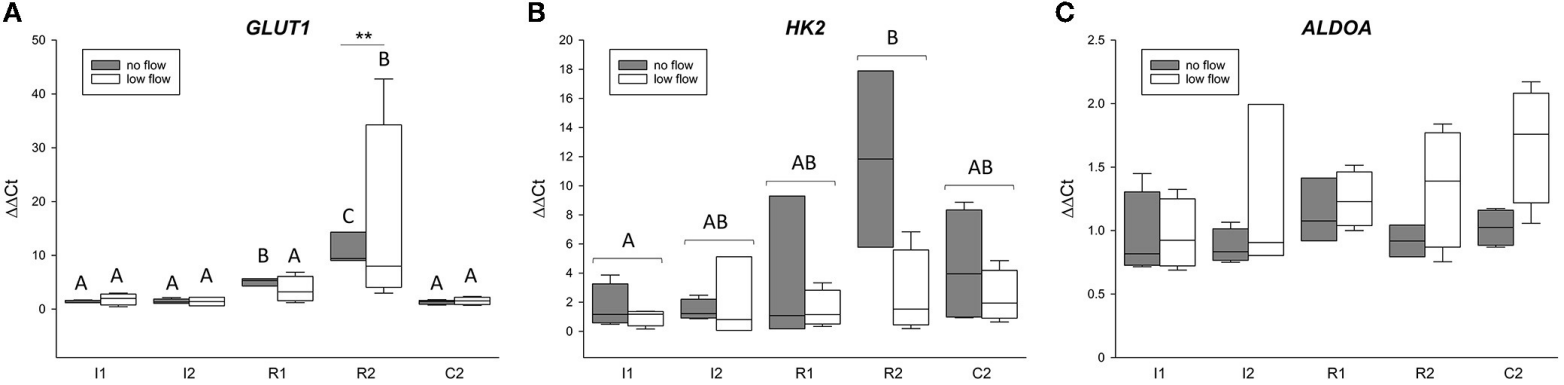
The mRNA expression of HIF1α target genes is upregulated in the equine jejunum epithelium after 1 and 2 h of ischemia and reperfusion, respectively. Gene expression of *GLUT1* **(A)**, *HK2* **(B)**, and *ALDOA* **(C)** is given relative to control tissue before onset of ischemia (C1), which was set to 1. Different letters indicate significant differences between the time points (*p* < 0.05, two-way RM ANOVA with *post-hoc* Holm-Sidak test). As there were interactions between time point and ischemia model for *GLUT1*, the comparison between time points is made individually for each group and asterisks indicate significant differences between the ischemia models at the same time point (**p < 0.01) for this gene. Otherwise, the comparison between the time points is made over both groups jointly as indicated by brackets. Boxes and error bars show the median, 10th, 25th, 75th, and 90th percentiles (no flow: gray; low flow: white); *N* = 5 (no flow) or 4 (low flow).

Figure 1 displays the gene expression of the HIF1α target genes *GLUT1, HK2*, and *ALDOA*, detailed statistical analysis of the mRNA expression analysis is given in Table 3. There was a statistically significant interaction of the factors time point and ischemia model for *GLUT1* (*p* < 0.05). The expression of *GLUT1* was significantly higher during reperfusion, i.e., in R1 and R2, compared to C2, I1, and I2 in the no flow group while in the low flow group GLUT1 expression in R2 significantly exceeded all other time points (*p* < 0.05). *HK2* was also increased over time and was significantly higher in R2 compared to I1 (*p* < 0.05). There was no interaction between time point and ischemia model for *HK2*. *ALDOA* did not change during the time course of the experiment but showed a significantly different expression between the ischemia models (p < 0.05). There was no interaction between the factors for *ALDOA*.

**Table 3:** Statistical data (*p*-values) for comparison of mRNA expression between time points I1, I2, R1, R2, and C2 and no flow vs. low flow model using a two-way RM ANOVA (with *post-hoc* Holm-Sidak test).

**Gene name**	**Time point**	**Ischemia model**	**Interactions**
*GLUT1*	**-**	-	**0.015**
*HK2*	**0.042**	0.111	0.180
*ALDOA*	0.310	**0.021**	0.419
*NRF2*	0.286	0.669	0.344
*G6PD*	0.746	0.102	0.618
*NQO1*	**0.041**	0.053	0.618
*SOD1*	**0.045**	**0.049**	0.669
*TXN2*	0.134	0.926	0.138
*PRDX3*	**0.018**	0.402	0.868
*PRDX5*	**0.002**	0.624	0.160
*GPX1*	**0.004**	0.554	0.574
*BNIP3*	**0.046**	0.894	0.297
*SOD2*	**<0.001**	0.349	0.137

**Figure 2:**
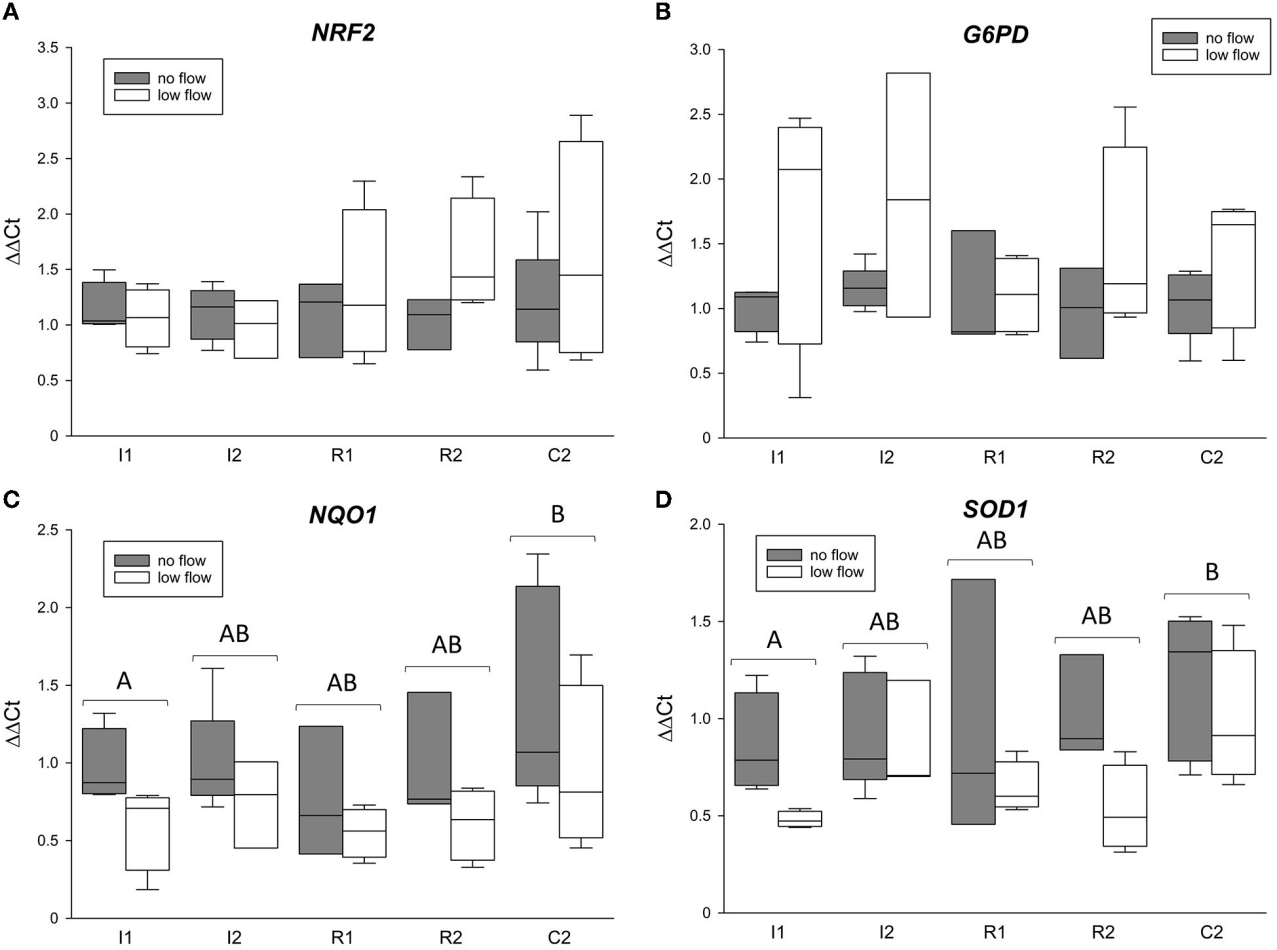
mRNA expression of *NRF2* **(A)** and its target genes *G6PD* **(B)**, *NQO1* **(C)**, and *SOD1* **(D)**. The gene expression is expressed relative to control tissue before the onset of ischemia (C1 = 1). Different letters indicate significant differences between the time points irrespective of the ischemia model (*p* < 0.05, two-way RM ANOVA with *post-hoc* Holm-Sidak test). Boxes and error bars show the median, 10th, 25th, 75th, and 90th percentiles (no flow: gray; low flow: white); *N* = 5 (no flow) or 4 (low flow).

**Figure 3:**
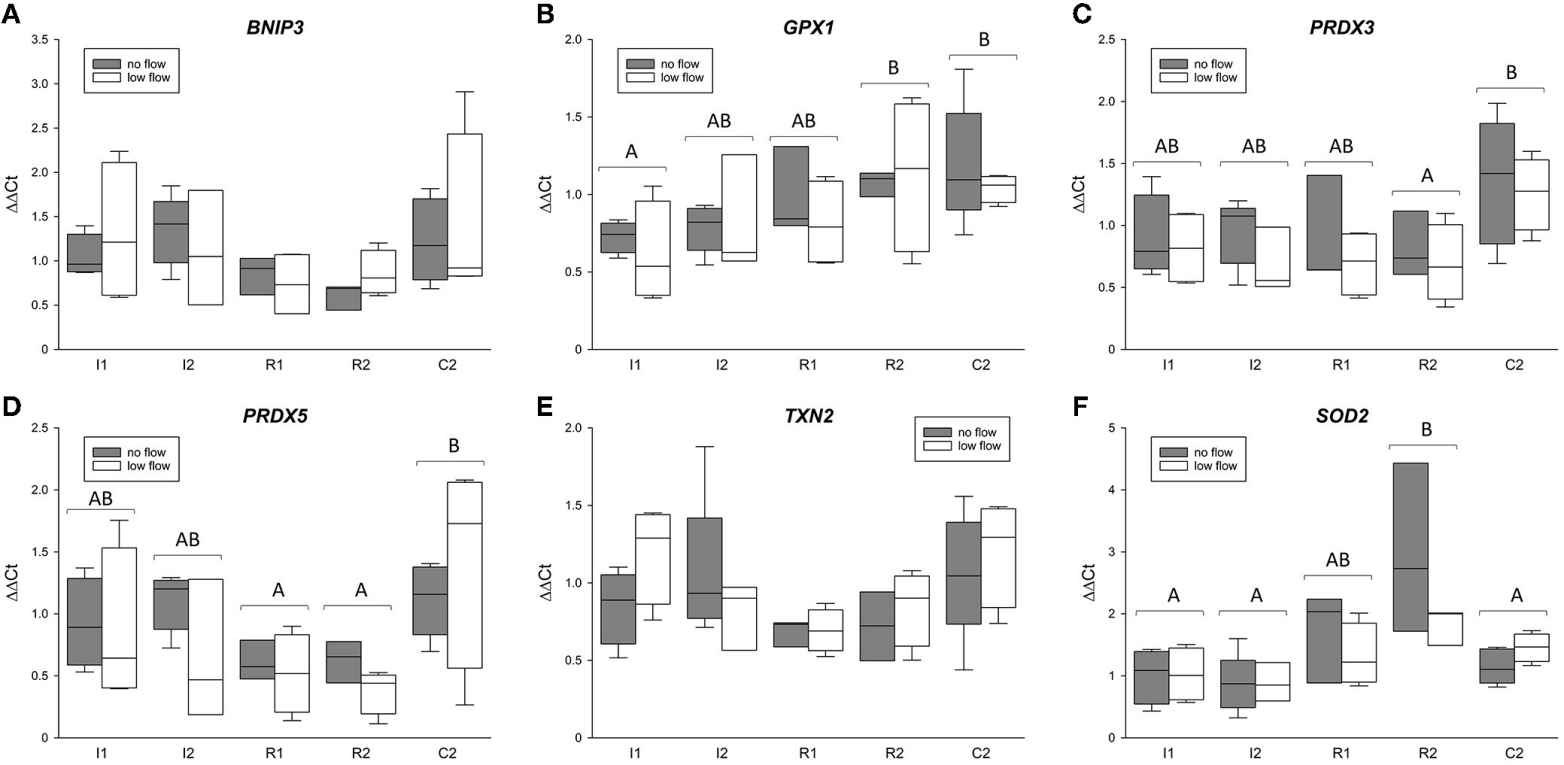
mRNA expression of *BNIP3* **(A)**, *GPX1* **(B)**, *PRDX3* **(C)** and *5* **(D)**, *TXN2* **(E)**, and *SOD2* **(F)** in the equine jejunum epithelium after 1 and 2 h of ischemia and reperfusion, respectively. The gene expression is given relative to control tissue before the onset of ischemia, which was set to 1. Different letters indicate significant differences between the time points irrespective of the ischemia model (*p* < 0.05, two-way RM ANOVA with *post-hoc* Holm-Sidak test). Boxes and error bars show the median, 10th, 25th, 75th, and 90th percentiles (no flow: gray; low flow: white); *N* = 5 (no flow) or 4 (low flow).

For detailed comparison see Figures 1–3. Statistically significant p-values are printed in bold.

Figure 2 shows the mRNA expression data for *NRF2, NQO1, G6PD*, and *SOD1. NRF2* and *G6PD* gene expression was not changed during the time course of the experiment and was not different between the groups (Figure 2 and Table 3). Both *NQO1* and *SOD1* mRNA expression was significantly increased in C2 compared to I1 (*p* < 0.05) but while *NQO1* did not differ between the ischemia models, *SOD1* was significantly different between low and no flow ischemia (*p* < 0.05). There were no interactions between time and ischemia model in any of these genes.

The gene expression for *TXN2, PRDX3* and *5, GPX1, BNIP3*, and *SOD2* is comprised in Figure 3. Except for *TXN2*, all of these genes changed significantly in the course of the experiment but showed no differences between the groups or interactions between ischemia model and time point. For *GPX1*, R2 and C2 significantly exceeded the expression in I1 (*p* < 0.05). *PRDX3* was significantly lower in R2 compared to C2 (*p* < 0.05). Similarly, *PRDX5* was significantly lower in R1 and R2 compared to C2 (*p* < 0.05). Regarding *SOD2*, R2 had a significantly higher mRNA expression compared to I1, I2, and C2 (*p* < 0.05).

### Western blot

**Figure 4:**
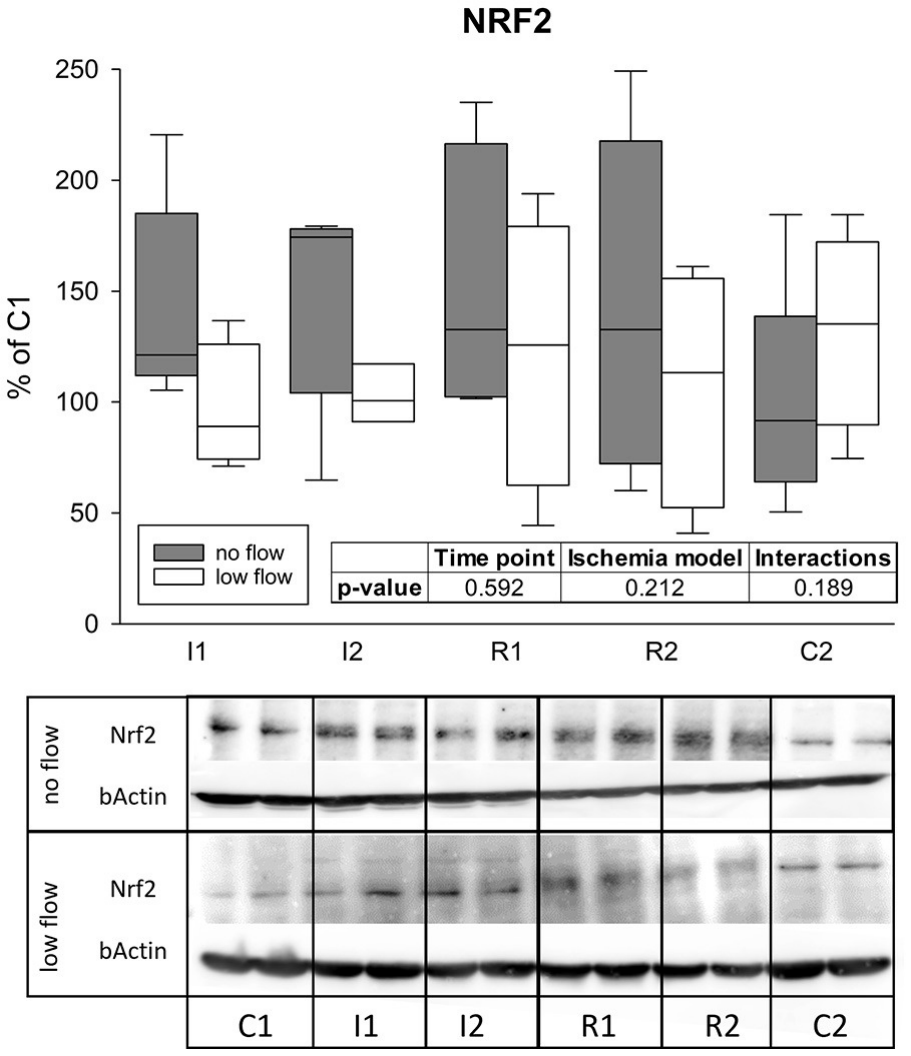
Protein levels of NRF2 are not changed during 1 and 2 h of ischemia and reperfusion each. Expression levels are given relative to C1 (=100%). Boxes and error bars show the median, 10th, 25th, 75th, and 90th percentiles (no flow: gray; low flow: white); *N* = 6 (no flow) or 4 (low flow). The insert shows the result (*p*-values) of the statistical analysis using a two-way RM ANOVA. Representative blots are shown below.

We found no significant changes in NRF2 protein levels during ischemia and reperfusion in any of the groups, differences between the groups or interactions of time point and ischemia model for NRF2 protein expression (Figure 4).

**Figure 5:**
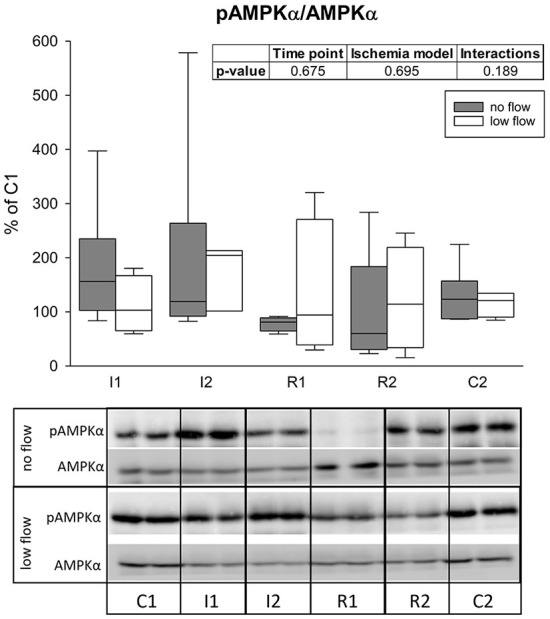
The ratio of pAMPKα/AMPK is not changed significantly during ischemia and reperfusion in equine jejunum epithelium. The amount of phosphorylated AMPKα in relation to its total level after 1 and 2 h of ischemia and reperfusion each is given relative to C1 (=100%). Boxes and error bars show the median, 10th, 25th, 75th, and 90th percentiles (no flow: gray; low flow: white); *N* = 6 (no flow) or 4 (low flow). The insert shows the result (*p*-values) of the statistical analysis using a two-way RM ANOVA. Representative blots are shown below.

The activation of AMPK was assessed as the ratio of pAMPKα to total AMPKα measured by western blot. Activation of AMPK could not be confirmed statistically and there were no differences between the groups or interactions (Figure 5).

**Figure 6:**
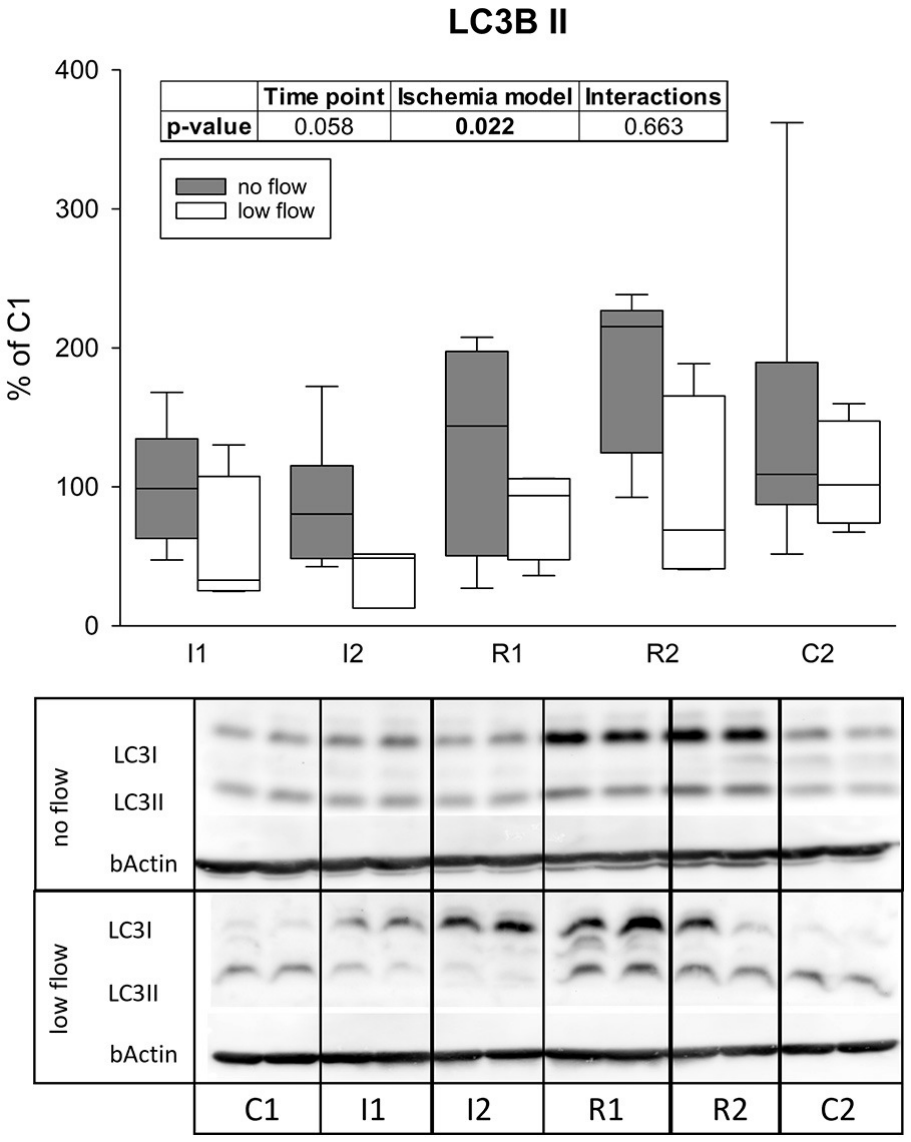
Levels of LC3II after 1 h and 2 h of ischemia and reperfusion each indicate a differing regulation of autophagy in no and low flow ischemia. Boxes and error bars show the median, 10th, 25th, 75th, and 90th percentiles (no flow: gray; low flow: white); *N* = 6 (no flow) or 4 (low flow). The insert shows the result (*p*-values) of the statistical analysis using a two-way RM ANOVA. Representative blots are shown below.

LC3II protein levels differed significantly between the ischemia models (*p* = 0.022) and tended to increase over time (*p* = 0.058), but there were no interactions between both factors (Figure 6).

### ROS levels

**Figure 7:**
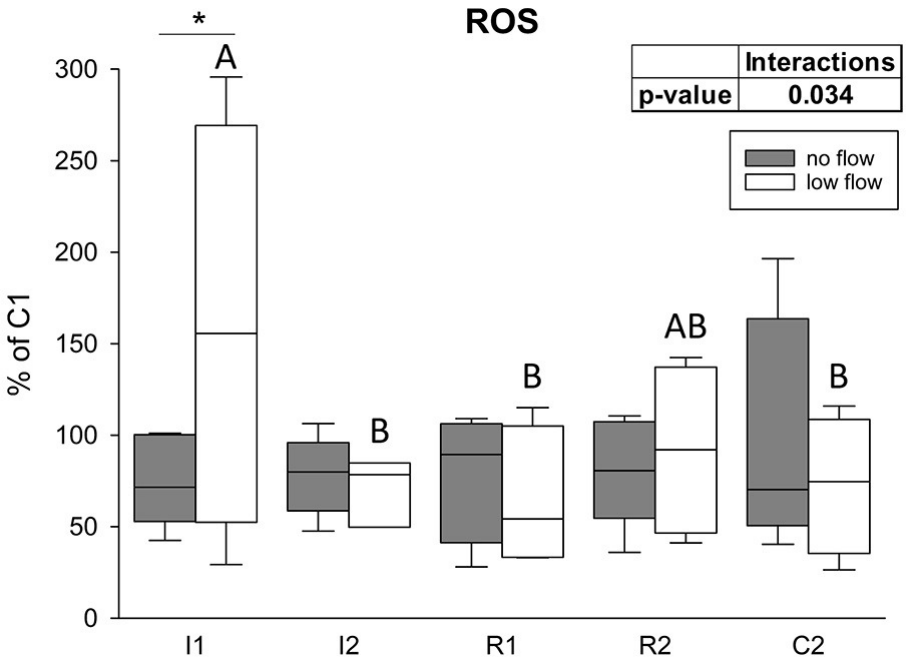
ROS levels are increased only after the onset of low flow ischemia in equine intestinal epithelium. Values are given relative to C1 (=100%, gray line). Asterisks indicate significant differences between the two groups at identical time points and different letters indicate significant differences between the time points in the low flow group exclusively (*p < 0.05, two-way RM ANOVA with *post-hoc* Holm-Sidak test). Boxes and error bars show the median, 10th, 25th, 75th, and 90th percentiles (no flow: gray; low flow: white); *N* = 5 (no flow) or *N* = 4 (low flow).

There was a significant interaction between the factors time point and ischemia model regarding ROS levels in the epithelium (*p* = 0.034). In the low flow group I1 significantly exceeded I2, R1, and C2, and it was also significantly different from the no flow group at this time point (Figure 7; *p* < 0.05). The no flow group showed no significant differences over time.

## Discussion

An efficient and timely adaptation to a lack of nutrients and oxygen is crucial for cellular survival and maintaining the epithelial integrity in intestinal IRI. AMPK, HIF1α, NRF2 as well as autophagy have been proposed to be main players mediating adaptation to ischemia and reperfusion in various tissues (48). All of them have been reported to interact with one another and to have both beneficial and detrimental impact on the outcome of IRI. This is the first study describing the involvement of these factors in equine jejunum epithelium after ischemia and reperfusion *in vivo*. By comparing low flow and no flow ischemia, interactions between the different pathways and their specific roles may be deduced and could be valuable targets for the development of (adjunct) therapies for equine colic. We hypothesized that during no flow ischemia HIF1α and AMPK are activated quickly due to the complete lack of oxygen and nutrients, whereas higher amounts of ROS formed in low flow ischemia rather stimulate antioxidative pathways such as NRF2. While we found substantial proof for an activation of HIF1α and increased autophagy, activation of AMPK could not be confirmed statistically and NRF2 seems to be of minor importance in the current model. However, similar pathways were activated during no and low flow ischemia although in varying intensities and timelines.

On mRNA expression level, our results indicate a stabilization of the master regulator of hypoxia, HIF1α, as indicated by the upregulation of its target genes *GLUT1* and *HK2*. The simultaneous development in the no and low flow group indicates that low flow ischemia is already sufficient to stabilize HIF1α. Interestingly, the ischemia models differed in the gene expression of *ALDOA*. This could be due to a remote ischemic preconditioning effect, i.e., an activation of the protective HIF1α pathway in remote segments by systemic signaling originating from the ischemic intestinal loops (49–51). This would be more likely to occur in low flow ischemia, considering this type of ischemia still permits entry of signaling molecules into the systemic circulation. This interpretation is supported by the differential expression of *ALDOA* in low and no flow ischemia.

On the protein level, AMPK is a fast adaptor to oxidative stress. It is not only activated by high AMP/ATP ratios, i.e., an energy deficit (21), but also by ROS, increased [Ca^2+^]_i_ or crosstalk with HIF1α (24). All these factors are likely present in the intestinal epithelium during IRI and especially in no flow ischemia. Accordingly, we observed a higher (although not significantly increased) pAMPKα/AMPKα ratio in the ischemic intestinal epithelium, which is in accordance to studies in the ischemic heart (37) and intestinal epithelial cells *in vitro* (27). Remarkably, the pAMPKα/AMPKα ratio is nearly doubled after 1 h of ischemia in the no flow group, whereas in the low flow group this increase happens only after 2 h of ischemia. None of these changes reached a statistically significant level, which might be due to the low number of biological replicates and high interindividual differences. It could be assumed that the complete occlusion of the mesenteric vessels in the no flow group led to low energy levels induced by oxygen and nutrient depletion more rapidly than in low flow ischemia, where a small portion of oxygen and nutrients could still reach the tissue.

Among other effects, AMPK is known to activate autophagy (22). It was previously reported, that the induction of autophagy exerted protective effects in IRI (25, 32, 36). While basal autophagy removes misfolded or surplus cell organelles, it is also an adaptive catabolic process in response to metabolic stress including nutrient deprivation and hypoxia, that supplies the cell with nutrients to balance the cellular energy requirements (52). During reperfusion, it removes damaged organelles and other cytotoxic agents (36). The best characterized marker for autophagy is LC3, which is associated with the autophagosome in its lipidated form LC3II (53, 54). In the present study, levels of LC3II differed significantly in no and low flow ischemia. While LC3II was slightly lower during ischemia in the low flow group compared to baseline, in the no flow group it was rather increased during reperfusion. Since LC3II is associated with the autophagosome and subsequently degraded in the autophagolysosome, decreased LC3II levels could indicate an increased degradation of autophagosomes, i.e., an increased autophagic flux. In line with this, the subsequent increase of LC3II in the no flow group may be a sign of an impaired autophagic flux, which has been described as a complication of increased autophagy of damaged organelles during IRI in other tissues (30, 36, 37). While our study was not intended to and cannot clarify the question whether autophagy is protective or harmful in equine intestinal IRI, it provides evidence that this evolutionary old and basic cellular process is activated during IRI in equine jejunum epithelium. In equine colon epithelium, an accumulation of autophagosomes was found in electron microscopy after IRI (55). Given that this was only a snapshot at the end of IRI, it is difficult to conclude what this means for the autophagic activity or flux, but it could support our interpretation of an impaired autophagic flux after reperfusion. As an accumulation of LC3II or autophagosomes could imply either an increased autophagic activity or an impaired autophagic flux (52, 53), the interpretation of these data should be considered with care. Additional analyses using transmission electron microscopy could have been helpful to gain further insights on the morphological level and are thus limiting the scope of the current study.

The part of the cell that is most affected by IRI are the mitochondria (56). Lack of oxygen, nutrients and subsequent metabolic changes quickly lead to mitochondrial dysfunction and damage. Damaged mitochondria are supposed to be the main sites of production of ROS during reoxygenation (36, 57). Due to the ischemic damage of the electron transport chain, the re-entry of oxygen also leads to a release of ROS from the mitochondria that induce subsequent cell damage (58). Accordingly, elimination of damaged mitochondria by mitophagy is the most important function of autophagy during the later phase of ischemia and reperfusion. The decreased mRNA expression of several mitochondrial markers such as *PRDX3* and *5* strongly supports the notion that mitophagy is activated in the equine jejunum epithelium during IRI and supports our interpretation of the LC3II protein levels.

While another aspect of the protection by AMPK might be the inhibition of ischemia induced opening of the mitochondrial permeability transition pore (25), thereby preventing ROS generation, other factors rather aim to scavenge ROS and attenuate oxidative stress. NRF2 is the master regulator of the antioxidative defense and controls the transcription of several hundred genes involved in redox homeostasis, cell metabolism and inflammation (41, 59, 60). It has been shown to be upregulated by ischemia and reperfusion in rat intestines and murine lungs (61, 62). Based on our findings on gene and protein expression level, we could not confirm activation of NRF2 in equine intestinal IRI. A lack of NRF2 activation may have resulted from an efficient removal of dysfunctional mitochondria and ROS, which is known as another important trigger for NRF2 activation (59).

In the current study, we found higher ROS levels in the low flow group at the beginning of ischemia and unchanged ROS levels during the following ischemia and reperfusion as well as in the no flow group. This is in contrast with the general opinion that ROS levels are elevated during ischemia and especially reperfusion, triggering cell damage and activation of adaptation mechanisms like HIF1α, AMPK, and NRF2 (33, 48). Our findings might be biased by the sampling method, storage duration or other methodological errors and we observed high interindividual differences between the horses. However, as we compared all samples to their own control, and potential degradation would have taken place in a similar dimension in these samples, the relative differences would remain unchanged. A possible explanation may be that the use of the volatile anesthetic isoflurane could have influenced the mitochondrial ROS production, although it has both been described to attenuate and enhance mitochondrial ROS generation (63, 64). Nevertheless, the role of ROS in equine intestinal IRI has been questioned long before. The levels of xanthine oxidase and dehydrogenase, the enzymes that are mainly involved in the generation of superoxide and attraction of neutrophils during reperfusion, are significantly lower in equine small intestine compared to rodent models in which the formation of ROS has mainly been investigated (65–69). Furthermore, the number of resident neutrophils and those migrating into the reperfused tissue are lower in horses than in other species (69). Therefore, not only the importance of ROS in but also the existence of reperfusion injury in equine colic is controversial (13, 70, 71). The divergent findings in the literature might originate from the implementation of different models, i.e., differences between low and no flow ischemia. In our study, there was no difference in ROS formation between the groups, except for the first hour of ischemia, when the low flow group had significantly elevated ROS levels. This might be explained by highly efficient scavenging of ROS in the equine intestinal epithelium. The increased mRNA levels of *SOD2*, even though this is the mitochondrial SOD isoform and all other mitochondrial transcription targets investigated were rather decreased, support this notion. The course of the SOD2 expression resembled our observations from the HIF1α target genes. Hence, we speculate that although SOD2 is a mitochondrial enzyme, it might be upregulated by HIF1α to enhance the antioxidative response. Lastly, highly efficient mitophagy, as indicated by our results, could have prevented the excessive liberation of ROS. Further experiments should be conducted to evaluate the actual production of ROS and to assess mitochondrial function in equine intestinal epithelium during the progression of IRI.

It must be mentioned that our study has some limitations due to the small number of biological replicates concurrently with a high interindividual variability. Thus, we can often only observe trends, while the differences between time points or ischemia models do not reach statistical significance. Nevertheless, these findings might have similar or even superior impact on our understanding of equine intestinal epithelial adaptation to IRI compared to *in vitro* experiments or experiments with laboratory rodents where a higher number of and also more homogenous replicates are easily available but may not represent the actual *in vivo* situation in equine intestinal epithelium.

In conclusion, we demonstrated that HIF1α is activated in IRI in the equine intestinal epithelium, depending on the intensity and duration of the ischemic insult, and apparently independently of the presence of ROS. Additionally, we found hints for an activation of AMPK, although this could not be verified statistically in our setup. These pathways orchestrate an adaptation of the epithelium by modulating the cellular metabolism and inducing auto- and especially mitophagy to prevent further harm to the enterocytes. Targeting these mechanisms may provide a novel approach for therapeutic strategies aiming to reduce intestinal IRI related mortality in horses and should be investigated in further detail. This study shows that the grade of ischemia can influence the adaptive mechanisms and provides data to assist in the choice of the ischemia model when investigating certain pathways. Moreover, our study demonstrates that it is essential to test different time points in the course of IRI to correctly evaluate the dynamic adaptation processes. Accordingly, categorizing adaptation mechanisms in beneficial and harmful based on endpoint analyses alone blocks our vision on the big picture.

## Data availability statement

The datasets presented in this study can be found in online repositories. The names of the repository/repositories and accession number(s) can be found in the article/Supplementary material.

## Ethics statement

The study was reviewed by the Ethics Committee for Animal Experiments of Lower Saxony, Germany, and approved according to Section 8 of the German Animal Welfare Act (LAVES 33.8-42502-04-19/3240).

## Author contributions

FD, FS, MG, and NV performed the experiments. FD, NV, and SK planned the study. FD wrote the manuscript. FD, FS, MG, NV, and SK revised the manuscript. All authors contributed to the article and approved the submitted version.

## Funding

FS was supported by the University of Veterinary Medicine Vienna Start-up Grant Number PP15018136.

## Conflict of interest

The authors declare that the research was conducted in the absence of any commercial or financial relationships that could be construed as a potential conflict of interest.

## Publisher's note

All claims expressed in this article are solely those of the authors and do not necessarily represent those of their affiliated organizations, or those of the publisher, the editors and the reviewers. Any product that may be evaluated in this article, or claim that may be made by its manufacturer, is not guaranteed or endorsed by the publisher.

## References

[1] van der Linden MA Laffont CM van Sloet Oldruitenborgh-Oosterbaan MM. Prognosis in equine medical and surgical colic. J Vet Intern Med. (2003) 17:343–8. 10.1111/j.1939-1676.2003.tb02459.x 12774977

[2] Moore RM Muir WW Granger DN. Mechanisms of gastrointestinal ischemia-reperfusion injury and potential therapeutic interventions: a review and its implications in the horse. J Vet Intern Med. (1995) 9:115–32. 10.1111/j.1939-1676.1995.tb03285.x 7674213

[3] Blikslager A Gonzalez L. Equine intestinal mucosal pathobiology. Annu Rev Anim Biosci. (2018) 6:157–75. 10.1146/annurev-animal-030117-014748 29144770 PMC7769316

[4] Mallick IH Yang W Winslet MC Seifalian AM. Ischemia-reperfusion injury of the intestine and protective strategies against injury. Dig Dis Sci. (2004) 49:1359–77. 10.1023/b:ddas.0000042232.98927.91 15481305

[5] Pascoe PJ McDonell WN Trim CM van Gorder J. Mortality rates and associated factors in equine colic operations - a retrospective study of 341 operations. Can Vet J. (1983) 24:76–85. 17422234 PMC1790318

[6] Kaufman JM Nekouei O Doyle AJ Biermann NM. Clinical findings, diagnoses, and outcomes of horses presented for colic to a referral hospital in Atlantic Canada (2000-2015). Can Vet J. (2020) 61:281–8. 32165752 PMC7020639

[7] Hunt JM Edwards GB Clarke KW. Incidence, diagnosis and treatment of postoperative complications in colic cases. Equine Vet J. (1986) 18:264–70. 10.1111/j.2042-3306.1986.tb03623.x 3758002

[8] Gerard MP Blikslager AT Roberts MC Tate LP Argenzio RA. The characteristics of intestinal injury peripheral to strangulating obstruction lesions in the equine small intestine. Equine Vet J. (1999) 31:331–5. 10.1111/j.2042-3306.1999.tb03826.x 10454093

[9] El-Malkey NF Alsemeh AE Ashour W Hassan NH Edrees HM. Fetuin-A exerts a protective effect against experimentally induced intestinal ischemia/reperfusion by suppressing autophagic cell death. Exp Biol Med. (2021) 246:1307–17. 10.1177/1535370221995207 33653159 PMC8371312

[10] VanderBroek AR Engiles JB Kästner SB Kopp V Verhaar N Hopster K. Protective effects of dexmedetomidine on small intestinal ischaemia-reperfusion injury in horses. Equine Vet J. (2021) 53:569–78. 10.1111/evj.13337 32862437

[11] Thoefner MB Ersbøll AK Jensen AL Hesselholt M. Factor analysis of the interrelationships between clinical variables in horses with colic. Prev Vet Med. (2001) 48:201–14. 10.1016/s0167-5877(00)00193-8 11182463

[12] Grootjans J Lenaerts K Buurman WA Dejong CH Derikx JP. Life and death at the mucosal-luminal interface: New perspectives on human intestinal ischemia-reperfusion. World J Gastroenterol. (2016) 22:2760–70. 10.3748/wjg.v22.i9.2760 26973414 PMC4777998

[13] Verhaar N Buhr N de Köckritz-Blickwede M von Hewicker-Trautwein M Pfarrer C Mazzuoli-Weber G. Ischaemic postconditioning reduces apoptosis in experimental jejunal ischaemia in horses. BMC Vet Res. (2021) 17:175. 10.1186/s12917-021-02877-y 33902575 PMC8077964

[14] Verhaar N Pfarrer C Neudeck S König K Rohn K Twele L. Preconditioning with lidocaine and xylazine in experimental equine jejunal ischaemia. Equine Vet J. (2021) 53:125–33. 10.1111/evj.13251 32119148

[15] Verhaar N Breves G Hewicker-Trautwein M Pfarrer C Rohn K Burmester M. The effect of ischaemic postconditioning on mucosal integrity and function in equine jejunal ischaemia. Equine Vet J. (2022) 54:427–37. 10.1111/evj.13450 34003501

[16] König KS Verhaar N Hopster K Pfarrer C Neudeck S Rohn K. Ischaemic preconditioning and pharmacological preconditioning with dexmedetomidine in an equine model of small intestinal ischaemia-reperfusion. PLoS ONE. (2020) 15:e0224720. 10.1371/journal.pone.0224720 32348301 PMC7190151

[17] Fandrey J Schödel J Eckardt K-U Katschinski DM Wenger RH. Now a Nobel gas: oxygen. Pflugers Arch. (2019) 471:1343–58. 10.1007/s00424-019-02334-8 31754831

[18] Semenza GL. Oxygen sensing, hypoxia-inducible factors, and disease pathophysiology. Annu Rev Pathol. (2014) 9:47–71. 10.1146/annurev-pathol-012513-104720 23937437

[19] Bauck AG Grosche A Morton AJ Graham AS Vickroy TW Freeman DE. Effect of lidocaine on inflammation in equine jejunum subjected to manipulation only and remote to intestinal segments subjected to ischemia. Am J Vet Res. (2017) 78:977–89. 10.2460/ajvr.78.8.977 28738006

[20] Ceulaer K de Delesalle C van Elzen R van Brantegem L Weyns A van Ginneken C. Morphological changes in the small intestinal smooth muscle layers of horses suffering from small intestinal strangulation. Is there a basis for predisposition for reduced contractility? Equine Vet J. (2011) 43:439–45. 10.1111/j.2042-3306.2010.00246.x 21496070

[21] Hardie DG. AMPK–sensing energy while talking to other signaling pathways. Cell Metab. (2014) 20:939–52. 10.1016/j.cmet.2014.09.013 25448702 PMC5693325

[22] Herzig S Shaw RJ. AMPK: guardian of metabolism and mitochondrial homeostasis. Nat Rev Mol Cell Biol. (2018) 19:121–35. 10.1038/nrm.2017.95 28974774 PMC5780224

[23] Mungai PT Waypa GB Jairaman A Prakriya M Dokic D Ball MK. Hypoxia triggers AMPK activation through reactive oxygen species-mediated activation of calcium release-activated calcium channels. Mol Cell Biol. (2011) 31:3531–45. 10.1128/MCB.05124-11 21670147 PMC3165558

[24] Dengler F. Activation of AMPK under hypoxia: many roads leading to Rome. Int J Mol Sci. (2020) 21:2428. 10.3390/ijms21072428 32244507 PMC7177550

[25] Qi D Young LH. AMPK: energy sensor and survival mechanism in the ischemic heart. Trends Endocrinol Metab. (2015) 26:422–9. 10.1016/j.tem.2015.05.010 26160707 PMC4697457

[26] Hayes HV Wolfe V O'Connor M Levinsky NC Piraino G Zingarelli B. Deficiency of AMPKα1 exacerbates intestinal injury and remote acute lung injury in mesenteric ischemia and reperfusion in mice. Int J Mol Sci. (2021) 22:9911. 10.3390/ijms22189911 34576076 PMC8468919

[27] Dengler F Gäbel G. The fast lane of hypoxic adaptation: glucose transport is modulated via a HIF-hydroxylase-AMPK-axis in jejunum epithelium. Int J Mol Sci. (2019) 20:4993. 10.3390/ijms20204993 31601024 PMC6834319

[28] Dengler F Rackwitz R Pfannkuche H Gäbel G. Glucose transport across lagomorph jejunum epithelium is modulated by AMP-activated protein kinase (AMPK) under hypoxia. J Appl Physiol. (1985) 123:1487–500. 10.1152/japplphysiol.00436.2017 28860168

[29] Haq S Grondin J Banskota S Khan WI. Autophagy: roles in intestinal mucosal homeostasis and inflammation. J Biomed Sci. (2019) 26:19. 10.1186/s12929-019-0512-2 30764829 PMC6375151

[30] Han X Tai H Wang X Wang Z Zhou J Wei X. AMPK activation protects cells from oxidative stress-induced senescence via autophagic flux restoration and intracellular NAD(+) elevation. Aging Cell. (2016) 15:416–27. 10.1111/acel.12446 26890602 PMC4854918

[31] Murrow L Debnath J. Autophagy as a stress-response and quality-control mechanism: implications for cell injury and human disease. Annu Rev Pathol. (2013) 8:105–37. 10.1146/annurev-pathol-020712-163918 23072311 PMC3971121

[32] Wen J Xu B Sun Y Lian M Li Y Lin Y. Paeoniflorin protects against intestinal ischemia/reperfusion by activating LKB1/AMPK and promoting autophagy. Pharmacol Res. (2019) 146:104308. 10.1016/j.phrs.2019.104308 31181335

[33] Wu M-Y Yiang G-T Liao W-T Tsai AP-Y Cheng Y-L Cheng P-W. Current mechanistic concepts in ischemia and reperfusion injury. Cell Physiol Biochem. (2018) 46:1650–67. 10.1159/000489241 29694958

[34] Zhang Y Liu D Hu H Zhang P Xie R Cui W. HIF-1α/BNIP3 signaling pathway-induced-autophagy plays protective role during myocardial ischemia-reperfusion injury. Biomed Pharmacother. (2019) 120:109464. 10.1016/j.biopha.2019.109464 31590128

[35] Mariño G Niso-Santano M Baehrecke EH Kroemer G. Self-consumption: the interplay of autophagy and apoptosis. Nat Rev Mol Cell Biol. (2014) 15:81–94. 10.1038/nrm3735 24401948 PMC3970201

[36] Ma S Wang Y Chen Y Cao F. The role of the autophagy in myocardial ischemia/reperfusion injury. Biochim Biophys Acta. (2015) 1852:271–6. 10.1016/j.bbadis.2014.05.010 24859226

[37] Hao M Zhu S Hu L Zhu H Wu X Li Q. Myocardial ischemic postconditioning promotes autophagy against ischemia reperfusion injury via the activation of the nNOS/AMPK/mTOR pathway. Int J Mol Sci. (2017) 18:614. 10.3390/ijms18030614 28287478 PMC5372630

[38] Huang Q Lou T Wang M Xue L Lu J Zhang H. Compound K inhibits autophagy-mediated apoptosis induced by oxygen and glucose deprivation/reperfusion via regulating AMPK-mTOR pathway in neurons. Life Sci. (2020) 254:117793. 10.1016/j.lfs.2020.117793 32416164

[39] Caleb I Erlitz L Telek V Vecsernyés M Sétáló G Hardi P. Characterizing autophagy in the cold ischemic injury of small bowel grafts: evidence from rat jejunum. Metabolites. (2021) 11:396. 10.3390/metabo11060396 34204418 PMC8234201

[40] Li Y Luo Y Li B Niu L Liu J Duan X. miRNA-182/Deptor/mTOR axis regulates autophagy to reduce intestinal ischaemia/reperfusion injury. J Cell Mol Med. (2020) 24:7873–83. 10.1111/jcmm.15420 32510855 PMC7348187

[41] Cadenas S. ROS and redox signaling in myocardial ischemia-reperfusion injury and cardioprotection. Free Radic Biol Med. (2018) 117:76–89. 10.1016/j.freeradbiomed.2018.01.024 29373843

[42] Vatistas NJ Snyder JR Nieto J Hildebrand SV Woliner MJ Harmon FA. Morphologic changes and xanthine oxidase activity in the equine jejunum during low flow ischemia and reperfusion. Am J Vet Res. (1998) 59:772–6. 9622750

[43] Hilton H Nieto JE Moore PF Harmon FA Naydan DK Snyder JR. Expression of cyclooxygenase genes in the jejunum of horses during low-flow ischemia and reperfusion. Am J Vet Res. (2011) 72:681–6. 10.2460/ajvr.72.5.681 21529221

[44] Reichert C Kästner SB Hopster K Rohn K Rötting AK. Use of micro-lightguide spectrophotometry for evaluation of microcirculation in the small and large intestines of horses without gastrointestinal disease. Am J Vet Res. (2014) 75:990–6. 10.2460/ajvr.75.11.990 25350089

[45] Xie F Xiao P Chen D Xu L Zhang B. miRDeepFinder: a miRNA analysis tool for deep sequencing of plant small RNAs. Plant Mol Biol. (2012) 80:75–84. 10.1007/s11103-012-9885-2 22290409

[46] Kehrer JP Paraidathathu T. The use of fluorescent probes to assess oxidative processes in isolated-perfused rat heart tissue. Free Radic Res Commun. (1992) 16:217–25. 10.3109/10715769209049175 1505782

[47] Ko S-F Chen K-H Wallace CG Yang C-C Sung P-H Shao P-L. Protective effect of combined therapy with hyperbaric oxygen and autologous adipose-derived mesenchymal stem cells on renal function in rodent after acute ischemia-reperfusion injury. Am J Transl Res. (2020) 12:3272–87. 32774699 PMC7407680

[48] Soares RO Losada DM Jordani MC Évora P Castro-E-Silva O. Ischemia/reperfusion injury revisited: an overview of the latest pharmacological strategies. Int J Mol Sci. (2019) 20:5034. 10.3390/ijms20205034 31614478 PMC6834141

[49] Albrecht M Zitta K Bein B Wennemuth G Broch O Renner J. Remote ischemic preconditioning regulates HIF-1α levels, apoptosis and inflammation in heart tissue of cardiosurgical patients: a pilot experimental study. Basic Res Cardiol. (2013) 108:314. 10.1007/s00395-012-0314-0 23203207

[50] Hummitzsch L Zitta K Berndt R Wong YL Rusch R Hess K. Remote ischemic preconditioning attenuates intestinal mucosal damage: insight from a rat model of ischemia-reperfusion injury. J Transl Med. (2019) 17:136. 10.1186/s12967-019-1885-4 31036020 PMC6489261

[51] Kant R Diwan V Jaggi AS Singh N Singh D. Remote renal preconditioning-induced cardioprotection: a key role of hypoxia inducible factor-prolyl 4-hydroxylases. Mol Cell Biochem. (2008) 312:25–31. 10.1007/s11010-008-9717-5 18273560

[52] Levine B Kroemer G. Autophagy in the pathogenesis of disease. Cell. (2008) 132:27–42. 10.1016/j.cell.2007.12.018 18191218 PMC2696814

[53] Klionsky DJ Abdel-Aziz AK Abdelfatah S Abdellatif M Abdoli A Abel S. Guidelines for the use and interpretation of assays for monitoring autophagy (4th edition)1. Autophagy. (2021) 17:1–382. 10.1080/15548627.2020.1797280 33634751 PMC7996087

[54] Zhang Z Singh R Aschner M. Methods for the detection of autophagy in mammalian cells. Curr Protoc Toxicol. (2016) 69:20.12.1–20.12.26. 10.1002/cptx.11 27479363 PMC4982470

[55] Grosche A Morton AJ Graham AS Sanchez LC Blikslager AT Polyak MM. Ultrastructural changes in the equine colonic mucosa after ischaemia and reperfusion. Equine Vet J Suppl. (2011) 43:8–15. 10.1111/j.2042-3306.2011.00402.x 21790749

[56] Kleinbongard P Skyschally A Heusch G. Cardioprotection by remote ischemic conditioning and its signal transduction. Pflugers Arch. (2017) 469:159–81. 10.1007/s00424-016-1922-6 27928644

[57] Kalogeris T Bao Y Korthuis RJ. Mitochondrial reactive oxygen species: a double edged sword in ischemia/reperfusion vs preconditioning. Redox Biol. (2014) 2:702–14. 10.1016/j.redox.2014.05.006 24944913 PMC4060303

[58] Seppet E Gruno M Peetsalu A Gizatullina Z Nguyen HP Vielhaber S. Mitochondria and energetic depression in cell pathophysiology. Int J Mol Sci. (2009) 10:2252–303. 10.3390/ijms10052252 19564950 PMC2695278

[59] Mata A Cadenas S. The antioxidant transcription factor Nrf2 in cardiac ischemia-reperfusion injury. Int J Mol Sci. (2021) 22:11939. 10.3390/ijms222111939 34769371 PMC8585042

[60] Bhattacharyya A Chattopadhyay R Mitra S Crowe SE. Oxidative stress: an essential factor in the pathogenesis of gastrointestinal mucosal diseases. Physiol Rev. (2014) 94:329–54. 10.1152/physrev.00040.2012 24692350 PMC4044300

[61] Jin C Fu W-L Zhang D-D Xing W-W Xia W-R Wei Z. The protective role of IL-1Ra on intestinal ischemia reperfusion injury by anti-oxidative stress via Nrf2/HO-1 pathway in rat. Biomed J. (2019) 42:36–45. 10.1016/j.bj.2018.11.001 30987703 PMC6468113

[62] Yan J Li J Zhang L Sun Y Jiang J Huang Y. Nrf2 protects against acute lung injury and inflammation by modulating TLR4 and Akt signaling. Free Radic Biol Med. (2018) 121:78–85. 10.1016/j.freeradbiomed.2018.04.557 29678610

[63] Zhang Y Xu Z Wang H Dong Y Shi HN Culley DJ. Anesthetics isoflurane and desflurane differently affect mitochondrial function, learning, and memory. Ann Neurol. (2012) 71:687–98. 10.1002/ana.23536 22368036 PMC3942786

[64] Wang J Sun J Qiao S Li H Che T Wang C. Effects of isoflurane on complex II-associated mitochondrial respiration and reactive oxygen species production: Roles of nitric oxide and mitochondrial KATP channels. Mol Med Rep. (2019) 20:4383–90. 10.3892/mmr.2019.10658 31545457

[65] Hartmann RM Licks F Schemitt EG Colares JR do Couto Soares M Zabot GP. Protective effect of glutamine on the main and adjacent organs damaged by ischemia-reperfusion in rats. Protoplasma. (2017) 254:2155–68. 10.1007/s00709-017-1102-3 28382390

[66] Wang A-L Niu Q Shi N Wang J Jia X-F Lian H-F. Glutamine ameliorates intestinal ischemia-reperfusion Injury in rats by activating the Nrf2/Are signaling pathway. Int J Clin Exp Pathol. (2015) 8:7896–904. 26339354 PMC4555682

[67] Tahir M Arshid S Fontes B Castro MS Luz IS Botelho KL. Analysis of the effect of intestinal ischemia and reperfusion on the rat neutrophils proteome. Front Mol Biosci. (2018) 5:89. 10.3389/fmolb.2018.00089 30555831 PMC6281993

[68] Gutiérrez-Sánchez G García-Alonso I Gutiérrez Sáenz de Santa María J Alonso-Varona A La Herrero de Parte B. Antioxidant-based therapy reduces early-stage intestinal ischemia-reperfusion injury in rats. Antioxidants. (2021) 10:853. 10.3390/antiox10060853 34071753 PMC8226848

[69] Blikslager AT Roberts MC Gerard MP Argenzio RA. How important is intestinal reperfusion injury in horses? J Am Vet Med Assoc. (1997) 211:1387–9. 9394886

[70] Laws EG Freeman DE. Significance of reperfusion injury after venous strangulation obstruction of equine jejunum. J Invest Surg. (1995) 8:263–70. 10.3109/08941939509031600 8519742

[71] Graham AS Grosche A Morton AJ Polyak MM Freeman DE. *In vitro* and *in vivo* responses of mucosa from the large colon of horses to ischemia and reperfusion. Am J Vet Res. (2011) 72:982–9. 10.2460/ajvr.72.7.982 21728860

